# *N*,*N*′-Bis(3-phenyl­prop-2-en-1-yl­idene)-2,2′-disulfanediyldianiline

**DOI:** 10.1107/S160053681004821X

**Published:** 2010-11-27

**Authors:** James Raftery, Sabina Jhaumeer-Laulloo, Minu G. Bhowon, Kiran Chikhooree, John A. Joule

**Affiliations:** a The School of Chemistry, The University of Manchester, Manchester M13 9PL, England; b Chemistry Department, University of Mauritius, Reduit, Mauritius

## Abstract

In the title compound, C_30_H_24_N_2_S_2_, the two phenyl rings attached to the S atoms are oriented nearly perpendicularly, making a dihedral angle of 86.14 (8)°. Each of the two ArCH=CHCH=N units is almost planar, having maximum deviations from the least-squares planes of 0.125 and 0.149 Å, and rotated around the C—N bonds relative to the adjacent phenyl ring by 110.26 and 30.30°.

## Related literature

The structure of the title compound was determined within a project on the synthesis of new ligands based on diaryl­disulfides, see: Bhowon *et al.* (2001 ▶, 2005 ▶, 2007 ▶); Raftery *et al.* (2009 ▶).
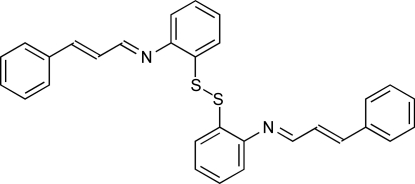


## Experimental

### 

#### Crystal data

C_30_H_24_N_2_S_2_
*M* *_r_* = 476.63
Monoclinic, 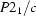

*a* = 20.2393 (13) Å
*b* = 9.1593 (6) Å
*c* = 13.5335 (8) Å
β = 104.995 (1)°
*V* = 2423.4 (3) Å^3^
*Z* = 4
Mo *K*α radiation
μ = 0.24 mm^−1^
*T* = 100 K
0.35 × 0.31 × 0.30 mm

#### Data collection

Bruker SMART CCD area-detector diffractometer
20381 measured reflections
5736 independent reflections
4962 reflections with *I* > 2σ(*I*)
*R*_int_ = 0.041

#### Refinement

*R*[*F* ^2^ > 2σ(*F* ^2^)] = 0.047
*wR*(*F* ^2^) = 0.112
*S* = 1.08
5736 reflections
307 parameters
H-atom parameters constrained
Δρ_max_ = 0.48 e Å^−3^
Δρ_min_ = −0.22 e Å^−3^

### 

Data collection: *SMART* (Bruker, 2007 ▶); cell refinement: *SAINT-Plus* (Bruker, 2007 ▶); data reduction: *SAINT-Plus*; program(s) used to solve structure: *SHELXS97* (Sheldrick, 2008 ▶); program(s) used to refine structure: *SHELXL97* (Sheldrick, 2008 ▶); molecular graphics: *SHELXTL* (Sheldrick, 2008 ▶); software used to prepare material for publication: *SHELXTL*.

## Supplementary Material

Crystal structure: contains datablocks I, global. DOI: 10.1107/S160053681004821X/nc2202sup1.cif

Structure factors: contains datablocks I. DOI: 10.1107/S160053681004821X/nc2202Isup2.hkl

Additional supplementary materials:  crystallographic information; 3D view; checkCIF report

## Figures and Tables

**Fig. 1.**
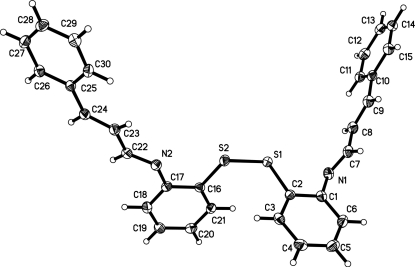
Crystal structure of the title compound with labelling and displacement elliposids drawn at the 50% probability level.

## supplementary crystallographic information

### Comment

The structure of the title compound was determined within a project on the synthesis of new ligands based on diaryldisulfides (Bhowon *et al.*, 2001; Bhowon *et al.* 2005; Bhowon *et al.*, 2007; Raftery *et al.* 2009). In this project we have synthesized 2,2'-dithiobis[*N*-(3-phenyl- 2-propen-1-ylidene) benzenamine via the condensation of 2,2'-dithiobis(benzenamine)  with cinnamaldehyde. The structure determination revealed that each of the ArCH=CHCH=N moieties are coplanar but rotated relative to the adjacent phenyl rings. Moreover, the phenyl rings attached to the sulfur atom are twisted around the sulfur-sulfur single bond so that they are nearly perpendicularly oriented.

### Experimental

2,2'-Dithiobis(benzenamine)(0.49 g, 2 mmol) was added to a solution of *trans* cinnamaldehyde (0.50 mL, 4 mmol) in ethanol (20 ml) and the mixture was heated at reflux for 3 h. On evaporation *in vacuo*, a yellow crude product was obtained which was recrystallised from CHCl_3_/Et_2_O to yield the bis-imine (86 percent) as yellow crystals, mp 435 K. I.R. 1624, 1609 cm^-1^; ^1^H-NMR (250 MHz, DMSO-*d*_6_) 8.33 (1H, d, 3 Hz), 8.31 (1H, d, 3 Hz), 7.66 (2H, dd, 4.5, 3 Hz), 7.59-7.62 (4H, m), 7.26-7.16 (2H, m), 7.05 (2H, dd, 4.5, 3 Hz). ^13^C-NMR (62.5 MHz, DMSO-*d*_6_) 163.1, 152.8, 148.8, 131.6, 129.8, 129.1, 129.0, 128.5, 128.0, 76.5. Anal. Calc. (Found) C, 75.4 (75.1), H, 5.0 (5.0), N, 5.9 (6.0), S, 13.9 (14.0).

### Refinement

H atoms were included in calculated positions with C—H distances of 0.95(CH), 0.99(CH_2_) & 0.98(CH_3_)Å; Uĩso(H) values were fixed at 1.2*U*_eq_(C) except for CH_3_ where Uĩso(H) values of 1.5*U*_eq_(C) were used.

### Crystal data

**Table:**

C_30_H_24_N_2_S_2_	*D*_x_ = 1.306 Mg m^−^^3^
*M**_r_* = 476.63	Melting point: 435 K
Monoclinic, *P*2_1_/*c*	Mo *K*α radiation, λ = 0.71073 Å
*a* = 20.2393 (13) Å	Cell parameters from 6928 reflections
*b* = 9.1593 (6) Å	θ = 2.5–28.3°
*c* = 13.5335 (8) Å	µ = 0.24 mm^−^^1^
β = 104.995 (1)°	*T* = 100 K
*V* = 2423.4 (3) Å^3^	Block, yellow
*Z* = 4	0.35 × 0.31 × 0.30 mm
*F*(000) = 1000	

### Data collection

**Table:**

Bruker SMART CCD area-detector diffractometer	4962 reflections with *I* > 2σ(*I*)
Radiation source: fine-focus sealed tube	*R*_int_ = 0.041
graphite	θ_max_ = 28.3°, θ_min_ = 2.1°
phi and ω scans	*h* = −26→26
20381 measured reflections	*k* = −12→12
5736 independent reflections	*l* = −17→17

### Refinement

**Table:**

Refinement on *F*^2^	Primary atom site location: structure-invariant direct methods
Least-squares matrix: full	Secondary atom site location: difference Fourier map
*R*[*F*^2^ > 2σ(*F*^2^)] = 0.047	Hydrogen site location: inferred from neighbouring sites
*wR*(*F*^2^) = 0.112	H-atom parameters constrained
*S* = 1.08	*w* = 1/[σ^2^(*F*_o_^2^) + (0.0595*P*)^2^ + 0.2559*P*] where *P* = (*F*_o_^2^ + 2*F*_c_^2^)/3
5736 reflections	(Δ/σ)_max_ = 0.001
307 parameters	Δρ_max_ = 0.48 e Å^−^^3^
0 restraints	Δρ_min_ = −0.22 e Å^−^^3^

### Special details

**Table:**

Geometry. All e.s.d.'s (except the e.s.d. in the dihedral angle between two l.s. planes) are estimated using the full covariance matrix. The cell e.s.d.'s are taken into account individually in the estimation of e.s.d.'s in distances, angles and torsion angles; correlations between e.s.d.'s in cell parameters are only used when they are defined by crystal symmetry. An approximate (isotropic) treatment of cell e.s.d.'s is used for estimating e.s.d.'s involving l.s. planes.
Refinement. Refinement of *F*^2^ against ALL reflections. The weighted *R*-factor *wR* and goodness of fit *S* are based on *F*^2^, conventional *R*-factors *R* are based on *F*, with *F* set to zero for negative *F*^2^. The threshold expression of *F*^2^ > σ(*F*^2^) is used only for calculating *R*-factors(gt) *etc*. and is not relevant to the choice of reflections for refinement. *R*-factors based on *F*^2^ are statistically about twice as large as those based on *F*, and *R*- factors based on ALL data will be even larger.

### Fractional atomic coordinates and isotropic or equivalent isotropic displacement parameters (Å^2^)

**Table:**

	*x*	*y*	*z*	*U*_iso_*/*U*_eq_	
C1	0.38459 (8)	1.25065 (18)	0.51729 (12)	0.0195 (3)	
C2	0.31992 (8)	1.18586 (17)	0.48098 (12)	0.0180 (3)	
C3	0.26430 (9)	1.23904 (19)	0.51183 (13)	0.0220 (3)	
H3	0.2206	1.1951	0.4875	0.026*	
C4	0.27272 (9)	1.3567 (2)	0.57839 (13)	0.0258 (4)	
H4	0.2347	1.3929	0.5998	0.031*	
C5	0.33636 (10)	1.4218 (2)	0.61399 (13)	0.0275 (4)	
H5	0.3416	1.5029	0.6592	0.033*	
C6	0.39234 (9)	1.36868 (19)	0.58369 (13)	0.0241 (4)	
H6	0.4359	1.4131	0.6084	0.029*	
C7	0.46740 (8)	1.24584 (19)	0.42592 (13)	0.0225 (4)	
H7	0.4539	1.3424	0.4040	0.027*	
C8	0.51906 (8)	1.17317 (19)	0.38739 (13)	0.0227 (4)	
H8	0.5395	1.0863	0.4198	0.027*	
C9	0.53883 (8)	1.22549 (19)	0.30712 (13)	0.0236 (4)	
H9	0.5202	1.3170	0.2809	0.028*	
C10	0.58617 (8)	1.15668 (19)	0.25538 (12)	0.0210 (3)	
C11	0.61820 (9)	1.02313 (19)	0.28774 (13)	0.0227 (4)	
H11	0.6085	0.9733	0.3439	0.027*	
C12	0.66411 (9)	0.9628 (2)	0.23856 (14)	0.0266 (4)	
H12	0.6864	0.8734	0.2622	0.032*	
C13	0.67738 (9)	1.03311 (19)	0.15495 (14)	0.0252 (4)	
H13	0.7087	0.9920	0.1213	0.030*	
C14	0.64471 (9)	1.1634 (2)	0.12084 (13)	0.0251 (4)	
H14	0.6532	1.2108	0.0629	0.030*	
C15	0.59981 (9)	1.22491 (19)	0.17048 (13)	0.0235 (4)	
H15	0.5780	1.3147	0.1466	0.028*	
C16	0.20139 (8)	0.85527 (17)	0.43037 (12)	0.0177 (3)	
C17	0.13867 (8)	0.78088 (17)	0.39865 (12)	0.0184 (3)	
C18	0.11973 (8)	0.68668 (18)	0.46757 (13)	0.0221 (4)	
H18	0.0778	0.6346	0.4471	0.026*	
C19	0.16135 (9)	0.66819 (19)	0.56546 (13)	0.0219 (3)	
H19	0.1476	0.6046	0.6119	0.026*	
C20	0.22306 (8)	0.74204 (18)	0.59609 (13)	0.0209 (3)	
H20	0.2515	0.7293	0.6634	0.025*	
C21	0.24311 (8)	0.83474 (18)	0.52809 (12)	0.0198 (3)	
H21	0.2857	0.8844	0.5487	0.024*	
C22	0.05392 (8)	0.72355 (18)	0.24995 (13)	0.0205 (3)	
H22	0.0494	0.6313	0.2796	0.025*	
C23	0.01189 (8)	0.75679 (18)	0.14901 (13)	0.0208 (3)	
H23	0.0135	0.8526	0.1229	0.025*	
C24	−0.02929 (8)	0.65861 (19)	0.09061 (12)	0.0204 (3)	
H24	−0.0340	0.5676	0.1217	0.025*	
C25	−0.06781 (8)	0.67666 (18)	−0.01621 (12)	0.0186 (3)	
C26	−0.10459 (8)	0.55894 (18)	−0.06838 (13)	0.0211 (3)	
H26	−0.1060	0.4698	−0.0331	0.025*	
C27	−0.13915 (9)	0.56984 (19)	−0.17104 (13)	0.0237 (4)	
H27	−0.1647	0.4892	−0.2051	0.028*	
C28	−0.13637 (9)	0.69832 (19)	−0.22380 (13)	0.0238 (4)	
H28	−0.1592	0.7054	−0.2944	0.029*	
C29	−0.09991 (8)	0.81704 (19)	−0.17271 (13)	0.0226 (4)	
H29	−0.0979	0.9053	−0.2087	0.027*	
C30	−0.06685 (8)	0.80699 (18)	−0.07039 (13)	0.0208 (3)	
H30	−0.0431	0.8894	−0.0360	0.025*	
N1	0.43975 (7)	1.18250 (16)	0.48865 (11)	0.0229 (3)	
N2	0.09713 (7)	0.81600 (15)	0.30036 (10)	0.0201 (3)	
S1	0.31958 (2)	1.03606 (5)	0.39689 (3)	0.02044 (11)	
S2	0.22014 (2)	0.97846 (5)	0.33968 (3)	0.02173 (11)	

### Atomic displacement parameters (Å^2^)

**Table:**

	*U*^11^	*U*^22^	*U*^33^	*U*^12^	*U*^13^	*U*^23^
C1	0.0200 (8)	0.0190 (8)	0.0186 (8)	0.0010 (6)	0.0031 (6)	0.0051 (6)
C2	0.0228 (8)	0.0156 (8)	0.0151 (7)	−0.0010 (6)	0.0043 (6)	0.0017 (6)
C3	0.0215 (8)	0.0226 (9)	0.0225 (8)	−0.0004 (7)	0.0068 (7)	0.0052 (7)
C4	0.0299 (9)	0.0242 (9)	0.0264 (9)	0.0043 (7)	0.0132 (7)	0.0020 (7)
C5	0.0382 (10)	0.0215 (9)	0.0227 (9)	−0.0001 (8)	0.0078 (8)	−0.0025 (7)
C6	0.0252 (9)	0.0220 (9)	0.0224 (9)	−0.0041 (7)	0.0012 (7)	0.0004 (7)
C7	0.0190 (8)	0.0228 (9)	0.0228 (9)	−0.0013 (6)	0.0005 (6)	0.0018 (7)
C8	0.0163 (8)	0.0256 (9)	0.0236 (9)	−0.0007 (6)	0.0004 (6)	0.0026 (7)
C9	0.0207 (8)	0.0242 (9)	0.0234 (9)	0.0006 (7)	0.0011 (7)	0.0019 (7)
C10	0.0166 (8)	0.0241 (9)	0.0192 (8)	−0.0030 (6)	−0.0009 (6)	−0.0010 (7)
C11	0.0240 (9)	0.0225 (9)	0.0183 (8)	−0.0016 (7)	−0.0005 (6)	0.0024 (7)
C12	0.0262 (9)	0.0233 (9)	0.0261 (9)	0.0031 (7)	−0.0010 (7)	−0.0007 (7)
C13	0.0224 (9)	0.0264 (9)	0.0258 (9)	−0.0007 (7)	0.0042 (7)	−0.0077 (7)
C14	0.0253 (9)	0.0280 (9)	0.0217 (9)	−0.0050 (7)	0.0053 (7)	−0.0003 (7)
C15	0.0223 (9)	0.0233 (9)	0.0229 (9)	0.0003 (7)	0.0027 (7)	0.0028 (7)
C16	0.0209 (8)	0.0162 (8)	0.0170 (8)	0.0002 (6)	0.0066 (6)	0.0004 (6)
C17	0.0186 (8)	0.0184 (8)	0.0174 (8)	0.0018 (6)	0.0035 (6)	−0.0013 (6)
C18	0.0194 (8)	0.0218 (8)	0.0250 (9)	−0.0020 (6)	0.0057 (7)	−0.0005 (7)
C19	0.0235 (9)	0.0213 (8)	0.0218 (8)	0.0008 (7)	0.0073 (7)	0.0044 (7)
C20	0.0220 (8)	0.0224 (8)	0.0166 (8)	0.0053 (6)	0.0016 (6)	0.0018 (6)
C21	0.0185 (8)	0.0198 (8)	0.0202 (8)	−0.0010 (6)	0.0034 (6)	−0.0021 (6)
C22	0.0210 (8)	0.0211 (8)	0.0204 (8)	−0.0016 (6)	0.0072 (7)	0.0006 (7)
C23	0.0205 (8)	0.0211 (8)	0.0207 (8)	−0.0008 (6)	0.0051 (6)	0.0006 (6)
C24	0.0199 (8)	0.0221 (8)	0.0194 (8)	−0.0019 (6)	0.0053 (6)	0.0016 (6)
C25	0.0145 (7)	0.0220 (8)	0.0195 (8)	−0.0002 (6)	0.0049 (6)	−0.0022 (6)
C26	0.0204 (8)	0.0201 (8)	0.0232 (8)	−0.0021 (6)	0.0061 (7)	0.0015 (7)
C27	0.0226 (9)	0.0235 (9)	0.0233 (9)	−0.0035 (7)	0.0027 (7)	−0.0047 (7)
C28	0.0218 (8)	0.0289 (9)	0.0185 (8)	0.0022 (7)	0.0011 (7)	−0.0003 (7)
C29	0.0207 (8)	0.0209 (8)	0.0260 (9)	0.0023 (6)	0.0058 (7)	0.0037 (7)
C30	0.0162 (8)	0.0197 (8)	0.0257 (9)	−0.0016 (6)	0.0037 (6)	−0.0029 (7)
N1	0.0183 (7)	0.0226 (7)	0.0273 (8)	−0.0009 (6)	0.0051 (6)	0.0010 (6)
N2	0.0190 (7)	0.0222 (7)	0.0184 (7)	−0.0008 (5)	0.0034 (5)	−0.0001 (6)
S1	0.0206 (2)	0.0211 (2)	0.0203 (2)	−0.00339 (15)	0.00639 (16)	−0.00296 (16)
S2	0.0230 (2)	0.0238 (2)	0.0160 (2)	−0.00575 (16)	0.00063 (16)	0.00236 (16)

### Geometric parameters (Å, °)

**Table:**

C1—C6	1.388 (2)	C16—C17	1.406 (2)
C1—C2	1.405 (2)	C16—S2	1.7790 (16)
C1—N1	1.418 (2)	C17—C18	1.395 (2)
C2—C3	1.386 (2)	C17—N2	1.415 (2)
C2—S1	1.7815 (16)	C18—C19	1.384 (2)
C3—C4	1.386 (2)	C18—H18	0.9500
C3—H3	0.9500	C19—C20	1.386 (2)
C4—C5	1.388 (3)	C19—H19	0.9500
C4—H4	0.9500	C20—C21	1.388 (2)
C5—C6	1.389 (2)	C20—H20	0.9500
C5—H5	0.9500	C21—H21	0.9500
C6—H6	0.9500	C22—N2	1.280 (2)
C7—N1	1.271 (2)	C22—C23	1.443 (2)
C7—C8	1.446 (2)	C22—H22	0.9500
C7—H7	0.9500	C23—C24	1.337 (2)
C8—C9	1.340 (2)	C23—H23	0.9500
C8—H8	0.9500	C24—C25	1.463 (2)
C9—C10	1.467 (2)	C24—H24	0.9500
C9—H9	0.9500	C25—C26	1.394 (2)
C10—C15	1.397 (2)	C25—C30	1.404 (2)
C10—C11	1.400 (2)	C26—C27	1.388 (2)
C11—C12	1.390 (2)	C26—H26	0.9500
C11—H11	0.9500	C27—C28	1.385 (2)
C12—C13	1.387 (3)	C27—H27	0.9500
C12—H12	0.9500	C28—C29	1.393 (2)
C13—C14	1.384 (2)	C28—H28	0.9500
C13—H13	0.9500	C29—C30	1.376 (2)
C14—C15	1.382 (2)	C29—H29	0.9500
C14—H14	0.9500	C30—H30	0.9500
C15—H15	0.9500	S1—S2	2.0302 (6)
C16—C21	1.386 (2)		
			
C6—C1—C2	119.62 (15)	C17—C16—S2	115.69 (12)
C6—C1—N1	123.68 (15)	C18—C17—C16	118.56 (15)
C2—C1—N1	116.53 (15)	C18—C17—N2	124.75 (15)
C3—C2—C1	120.12 (15)	C16—C17—N2	116.54 (14)
C3—C2—S1	126.42 (13)	C19—C18—C17	120.73 (15)
C1—C2—S1	113.46 (12)	C19—C18—H18	119.6
C4—C3—C2	119.72 (16)	C17—C18—H18	119.6
C4—C3—H3	120.1	C18—C19—C20	120.31 (15)
C2—C3—H3	120.1	C18—C19—H19	119.8
C3—C4—C5	120.45 (16)	C20—C19—H19	119.8
C3—C4—H4	119.8	C19—C20—C21	119.72 (15)
C5—C4—H4	119.8	C19—C20—H20	120.1
C4—C5—C6	120.11 (16)	C21—C20—H20	120.1
C4—C5—H5	119.9	C16—C21—C20	120.36 (15)
C6—C5—H5	119.9	C16—C21—H21	119.8
C1—C6—C5	119.98 (16)	C20—C21—H21	119.8
C1—C6—H6	120.0	N2—C22—C23	121.28 (15)
C5—C6—H6	120.0	N2—C22—H22	119.4
N1—C7—C8	121.00 (16)	C23—C22—H22	119.4
N1—C7—H7	119.5	C24—C23—C22	122.86 (16)
C8—C7—H7	119.5	C24—C23—H23	118.6
C9—C8—C7	121.56 (16)	C22—C23—H23	118.6
C9—C8—H8	119.2	C23—C24—C25	126.82 (16)
C7—C8—H8	119.2	C23—C24—H24	116.6
C8—C9—C10	127.03 (16)	C25—C24—H24	116.6
C8—C9—H9	116.5	C26—C25—C30	118.10 (15)
C10—C9—H9	116.5	C26—C25—C24	119.41 (15)
C15—C10—C11	118.30 (16)	C30—C25—C24	122.43 (15)
C15—C10—C9	119.53 (15)	C27—C26—C25	121.07 (16)
C11—C10—C9	122.17 (15)	C27—C26—H26	119.5
C12—C11—C10	120.67 (16)	C25—C26—H26	119.5
C12—C11—H11	119.7	C28—C27—C26	119.99 (16)
C10—C11—H11	119.7	C28—C27—H27	120.0
C13—C12—C11	120.05 (16)	C26—C27—H27	120.0
C13—C12—H12	120.0	C27—C28—C29	119.62 (16)
C11—C12—H12	120.0	C27—C28—H28	120.2
C14—C13—C12	119.71 (17)	C29—C28—H28	120.2
C14—C13—H13	120.1	C30—C29—C28	120.28 (16)
C12—C13—H13	120.1	C30—C29—H29	119.9
C15—C14—C13	120.45 (16)	C28—C29—H29	119.9
C15—C14—H14	119.8	C29—C30—C25	120.91 (15)
C13—C14—H14	119.8	C29—C30—H30	119.5
C14—C15—C10	120.80 (16)	C25—C30—H30	119.5
C14—C15—H15	119.6	C7—N1—C1	119.70 (15)
C10—C15—H15	119.6	C22—N2—C17	120.57 (14)
C21—C16—C17	120.31 (14)	C2—S1—S2	106.46 (6)
C21—C16—S2	123.95 (12)	C16—S2—S1	105.61 (6)
			
C6—C1—C2—C3	−0.4 (2)	C17—C18—C19—C20	−0.8 (3)
N1—C1—C2—C3	175.08 (15)	C18—C19—C20—C21	−0.1 (3)
C6—C1—C2—S1	−179.93 (12)	C17—C16—C21—C20	−0.8 (2)
N1—C1—C2—S1	−4.46 (19)	S2—C16—C21—C20	176.64 (12)
C1—C2—C3—C4	0.2 (2)	C19—C20—C21—C16	0.9 (2)
S1—C2—C3—C4	179.67 (13)	N2—C22—C23—C24	173.89 (16)
C2—C3—C4—C5	0.2 (3)	C22—C23—C24—C25	−172.68 (15)
C3—C4—C5—C6	−0.5 (3)	C23—C24—C25—C26	175.30 (16)
C2—C1—C6—C5	0.1 (2)	C23—C24—C25—C30	−1.8 (3)
N1—C1—C6—C5	−174.99 (16)	C30—C25—C26—C27	0.2 (2)
C4—C5—C6—C1	0.3 (3)	C24—C25—C26—C27	−177.00 (15)
N1—C7—C8—C9	166.62 (17)	C25—C26—C27—C28	1.2 (3)
C7—C8—C9—C10	−174.57 (16)	C26—C27—C28—C29	−1.3 (3)
C8—C9—C10—C15	178.26 (17)	C27—C28—C29—C30	−0.1 (3)
C8—C9—C10—C11	−1.3 (3)	C28—C29—C30—C25	1.5 (2)
C15—C10—C11—C12	2.1 (2)	C26—C25—C30—C29	−1.6 (2)
C9—C10—C11—C12	−178.36 (16)	C24—C25—C30—C29	175.55 (15)
C10—C11—C12—C13	−1.6 (3)	C8—C7—N1—C1	−173.79 (15)
C11—C12—C13—C14	0.0 (3)	C6—C1—N1—C7	−74.5 (2)
C12—C13—C14—C15	1.0 (3)	C2—C1—N1—C7	110.26 (18)
C13—C14—C15—C10	−0.5 (3)	C23—C22—N2—C17	−179.07 (15)
C11—C10—C15—C14	−1.1 (2)	C18—C17—N2—C22	−30.3 (2)
C9—C10—C15—C14	179.36 (15)	C16—C17—N2—C22	154.31 (15)
C21—C16—C17—C18	0.0 (2)	C3—C2—S1—S2	8.01 (16)
S2—C16—C17—C18	−177.67 (12)	C1—C2—S1—S2	−172.48 (10)
C21—C16—C17—N2	175.68 (14)	C21—C16—S2—S1	13.04 (15)
S2—C16—C17—N2	−1.99 (19)	C17—C16—S2—S1	−169.38 (11)
C16—C17—C18—C19	0.8 (2)	C2—S1—S2—C16	−86.14 (8)
N2—C17—C18—C19	−174.51 (15)		
